# Deep-learning approach to detect childhood glaucoma based on periocular photograph

**DOI:** 10.1038/s41598-023-37389-2

**Published:** 2023-06-22

**Authors:** Yoshiyuki Kitaguchi, Rina Hayakawa, Rumi Kawashima, Kenji Matsushita, Hisashi Tanaka, Ryo Kawasaki, Takahiro Fujino, Shinichi Usui, Hiroshi Shimojyo, Tomoyuki Okazaki, Kohji Nishida

**Affiliations:** 1 Department of Ophthalmology, Osaka University Graduate School of Medicine, 2-2 Yamadaoka, Suita, Osaka 565-0871 Japan; 2 Division of Health Science, Department of Medical Physics and Engineering, Osaka University Graduate School of Medicine, Osaka, Japan; 3 Artificial Intelligence Center for Medical Research and Application, Osaka University Hospital, Suita, Osaka Japan; 4 Institute for Open and Transdisciplinary Research Initiatives (OTRI), Osaka University, Suita, Osaka Japan

**Keywords:** Eye diseases, Medical research

## Abstract

Childhood glaucoma is one of the major causes of blindness in children, however, its diagnosis is of great challenge. The study aimed to demonstrate and evaluate the performance of a deep-learning (DL) model for detecting childhood glaucoma based on periocular photographs. Primary gaze photographs of children diagnosed with glaucoma with appearance features (corneal opacity, corneal enlargement, and/or globe enlargement) were retrospectively collected from the database of a single referral center. DL framework with the RepVGG architecture was used to automatically recognize childhood glaucoma from photographs. The average receiver operating characteristic curve (AUC) of fivefold cross-validation was 0.91. When the fivefold result was assembled, the DL model achieved an AUC of 0.95 with a sensitivity of 0.85 and specificity of 0.94. The DL model showed comparable accuracy to the pediatric ophthalmologists and glaucoma specialists in diagnosing childhood glaucoma (0.90 vs 0.81, p = 0.22, chi-square test), outperforming the average of human examiners in the detection rate of childhood glaucoma in cases without corneal opacity (72% vs. 34%, p = 0.038, chi-square test), with a bilateral corneal enlargement (100% vs. 67%, p = 0.03), and without skin lesions (87% vs. 64%, p = 0.02). Hence, this DL model is a promising tool for diagnosing missed childhood glaucoma cases.

## Introduction

Childhood glaucoma accounts for 4–18% of all causes of blindness in children^1–3^. This disease includes two types of pathogenesis: primary congenital glaucoma associated with primary maldevelopment of the trabecular meshwork and secondary glaucoma, in which aqueous outflow is reduced due to other ocular or systemic diseases^4,5^. Elevated intraocular pressure (IOP) leads to visual dysfunction by damaging the optic nerve and causing corneal scarring^1–4^. It also causes cosmetic disfigurement such as globe enlargement (buphthalmos) and corneal opacity (hydrophthalmos)^6^. To avoid these irreversible sequelae, it is important to identify the affected children at an early stage.

The diagnosis of childhood glaucoma involves multiple diagnostic methods, such as tonometry, funduscopy, and gonioscopy^7–10^. In some cases, general anesthesia is required because children may not cooperate during these exams^10^. Identification of any external symptoms is crucial as the first step in the diagnostic process to motivate performing these exams. Childhood glaucoma is characterized by three typical signs: photophobia, blepharospasm, and tearing; however, these non-specific signs may be absent or misinterpreted by parents, caregivers, and primary physicians^7–10^. Furthermore, mild and bilateral enlargement of the globes or corneal opacities may go unnoticed, resulting in delayed diagnoses and consultations with childhood glaucoma specialists for months or even years^11^.

In the last decade, advances have been made in the use of deep learning to detect pediatric ophthalmic diseases. Deep-learning methods, such as deep convolutional neural networks (DCNNs), have achieved high accuracy in the detection of strabismus^12^, leukocoria^13^, and retinopathy of prematurity, comparable to that of specialists^14^. However, systems specifically designed to screen for childhood glaucoma have not been reported. A deep learning model for detecting childhood-glaucoma-associated periocular appearance changes may be expected to increase access to specialists and speed up diagnosis.

In this study, we present deep-learning algorithms that detect childhood glaucoma using distinctive facial features that potentially contribute to the automated screening of the disease.

## Related work

The main machine learning algorithm used for diagnosing rare diseases from facial photos has changed with the growth of image analysis technology. In the early 2010s, the primary approach was to use hand-crafted features and multinomial linear regression, which were successful in distinguishing 14 genetic disorders from 202 facial photographs with an accuracy of up to 60%^14^. The implementation of automated facial landmark detectors, such as OpenCV and Dlib, along with machine learning algorithms like Support Vector Machines and k-Nearest Neighbors, led to an increase in prediction accuracy^15–17^. In the late 2010s, the use of end-to-end deep learning using convolutional networks improved both accuracy and processing speed, and in some cases outperformed human experts in diagnosing genetic diseases in binary tasks^18^. More recently, fine-tuning pre-trained Convolutional Neural Networks has led to even higher accuracy, even with limited amounts of training data^19–21^.

Several deep learning models have been reported for diagnosing glaucoma based on fundus findings, particularly optic nerve head findings^22–24^.

## Results

### Dataset characteristics

We identified 62 childhood glaucoma from our medical records. Of these patients, 231 images from 35 patients (11 patients with congenital glaucoma and 24 patients with secondary glaucoma) were included in the glaucoma group with the characteristic facial features described above. The background of secondary glaucoma comprised anterior segment dysgenesis (eight patients), retinopathy of prematurity (five patients), Sturge–Weber syndrome (five patients), persistent hyperplastic primary vitreous, Stickler syndrome, neurofibromatosis, juvenile xanthogranuloma, and Rubinstein–Taybi syndrome (one patient each). We selected 693 images from 693 patients as the controls. The number of patients in each category is listed in Table 1. Among all eligible patients, the distribution of age, sex, and eye position were not significantly different between glaucoma and control groups.

**Table 1:** Distribution of age, sex, and eye position between cases and controls.

		Childhood glaucoma	Controls	p-value
Number of subjects		35	690	
Number of images		231	693	
Age	Years (standard deviation)	3.4 ± 2.8*	3.5 ± 2.7*	0.63*
Sex	Male [per image] (%)	18 [121^a^] (2.4% [13.1%])	339 [339^a^] (46.6% [36.6%])	0.36^a,^^#^
	Female [per image] (%)	17 [110^a^] (2.3% [12.0%])	354 [354^a^] (48.6% [38.3%])	
Eye position	Esotropia (%)	20 (2.2%^#^)	60 (6.5%^#^)	0.99^a,#^
	Orthotropic (%)	129 (14.0%^#^)	387 (41.8%^#^)	
	Exotropia (%)	81 (8.8%^#^)	246 (26.7%^#^)	

P < 0.05 was recognized as a statistical difference.

^a^Number of total images.

*Welch t-test (age).

^#^Chi-square test (sex and eye position).

In this study, 27 patients per 65 identified patients (65%) were excluded based on the following exclusion criteria: lack of corneal opacity and corneal enlargement due to onset after the age of 4 years or successful control of IOP with topical medication. Other major reasons included retinopathy of prematurity, which resulted in phthisis bulbi following the diagnosis of secondary glaucoma.

### Performance of deep-learning model for diagnosing childhood glaucoma

**Figure 1:**
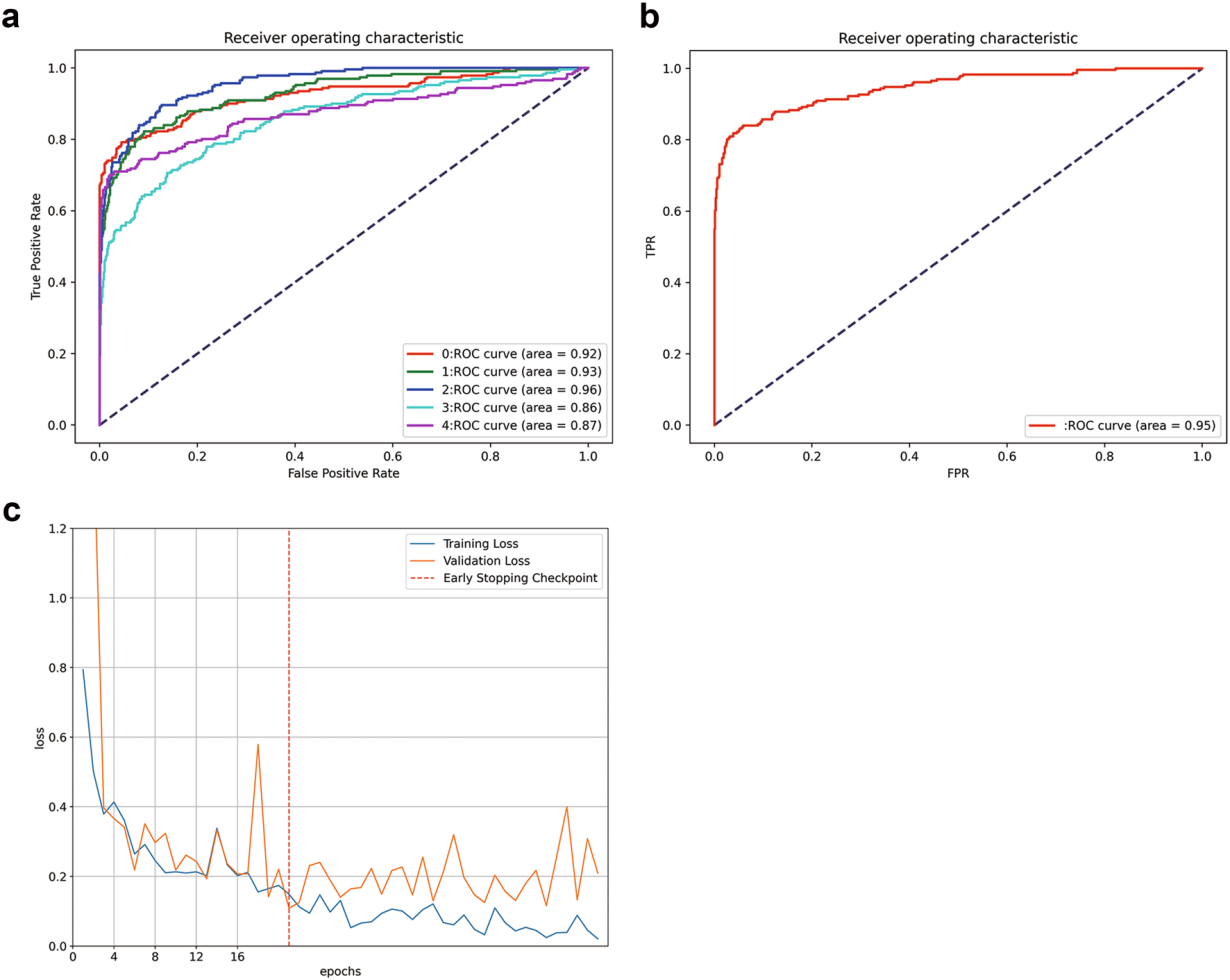
The receiver operating characteristic (ROC) curves of **(a)** each fivefold by the deep learning algorithms and **(b)** the ensembled prediction when a positive result at least onefold was defined as positive. **(c)** Typical training curve of the deep learning model. The training accuracy and validating accuracy are plotted against epochs.

In fivefold cross-validation, the deep-learning model showed a predictive accuracy of 87% (95% confidence interval [CI] 81–93%); sensitivity was 0.75 (95% CI 0.68–0.84), specificity was 0.90 (95% CI 0.82–0.99), positive predictive value was 0.74 (95% CI 0.67–0.81), f-measure was 0.74 (95% CI 0.67–0.81) and AUC was 0.91 (95% CI 0.87–0.94) (Fig. 1A). Ensemble of 5-folds improved AUC to 0.95 (Table 2, Fig. 1B). A representative learning curve is shown in Fig. 1C.

**Table 2:** The performance of deep learning model for diagnosing childhood glaucoma using fivefold cross-validation.

	Diagnostic performance					
	Accuracy	Sensitivity	Specificity	Positive Predictive value	F-measure	AUC
Fivefold average (95% CI)	0.87 (0.81–0.93)	0.75 (0.68–0.84)	0.90 (0.82–0.99)	0.75 (0.63–0.88)	0.74 (0.67–0.81)	0.91 (0.87–0.94)
Ensemble result	0.88	0.85	0.94	0.73	0.79	0.95

*CI* confidence interval.

### Interpretability of the deep-learning system

**Figure 2:**
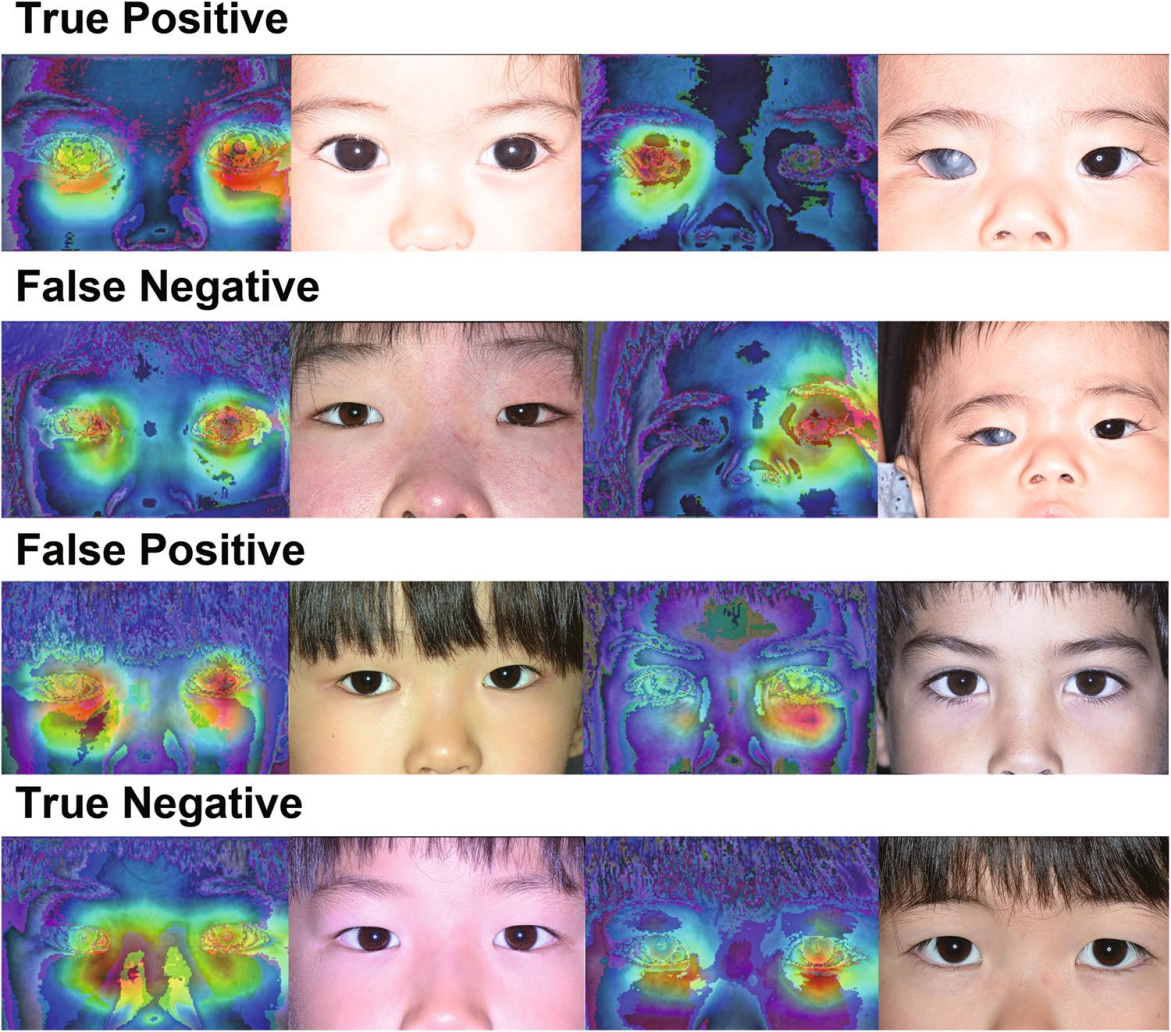
Examples of images and corresponding GradCAM heatmaps of childhood glaucoma and control patients.

Grad-CAM saliency maps showed that attention was distributed in the periocular area, including the cornea, bilaterally or unilaterally in both glaucoma and control groups (Fig. 2). In unilateral cases, the attention was mostly on the affected side; however, in a few cases in which the attention was on the unaffected side were misjudged as controls (Fig. 2).

### Comparison of the deep-learning model and ophthalmologist diagnosis

The overall accuracy of the deep-learning model was comparable to that of pediatric ophthalmologists and glaucoma specialists (Table 3, p = 0.22, chi-squared test). Table 4 shows the detection rates of childhood glaucoma using the deep-learning model and human graders disaggregated by periocular features. Human graders showed a lower detection rate of childhood glaucoma in images without corneal opacity than in those with corneal opacity (15/44, (34%) vs. 76/96 (79%), p < 0.001, Fisher’s exact test), whereas deep-learning showed a good detection rate in both images with corneal opacity and without corneal opacity (23/24 (96%) vs. 11/14 (72%), p = 0.132, Fisher’s exact test). The deep-learning model showed a higher detection rate of childhood glaucoma compared to human examiners for images without corneal opacity (8/11 (72%) vs. 15/44 (34%), p = 0.04), bilateral corneal enlargement (12/12 (100%) vs. 32/48 (67%), p = 0.03), and without skin lesions (27/31 (87%) vs. 79/124 (64%), p = 0.02).

**Table 3:** The diagnostic performance of deep learning model and human graders.

	Accuracy	Sensitivity	Specificity
DL model	0.90*	0.86	0.91
Human graders			
Pediatric ophthalmologist #1	0.88	0.86	0.91
Pediatric ophthalmologist #2	0.80	0.63	0.97
Glaucoma specialist #1	0.81	0.63	1.00
Glaucoma specialist #2	0.74	0.49	1.00
Average ± standard deviation	0.81 ± 0.05*	0.65 ± 0.13	0.97 ± 0.04

*DL* deep learning.

*P = 0.22, chi-square test (deep-learning model vs. average of human graders).

**Table 4:** Relationship of cosmetic features to the detection rate by deep learning model and human graders.

		DL model	Human graders	p-value*
Corneal opacity	Yes	23/24 (96%)	76/96 (79%)	0.07
	No	8/11 (72%)	15/44 (34%)	0.04
Corneal enlargement	Bilateral	12/12 (100%)	32/48 (67%)	0.03
	Unilateral	19/23 (83%)	59/92 (64%)	0.13
Skin lesion (Sturge–Weber syndrome)	Yes	4/4 (100%)	12/16 (75%)	0.54
	No	27/31 (87%)	79/124 (64%)	0.02

Diagrams of image dataset collection and pre-processing for detecting childhood glaucoma using deep learning.

*DL* deep learning, *TP* true positive, *FN* false negative.

*Fisher’s exact test (DL model vs. average of human graders).

## Discussion

We present the first automated facial diagnostic system for the detection of childhood glaucoma using a deep-learning model. This system uses a convolutional neural network based on transfer learning from a large-scale pretraining dataset to extract facial features. The system achieved comparatively high accuracy in the binary classification of childhood glaucoma by pediatric ophthalmologists and glaucoma specialists.

Visual inspection plays an important role in the diagnosis of glaucoma in childhood^10^. Missing symptoms inevitably delay diagnosis and treatment, resulting in amblyopia and a worse visual prognosis^25,26^. One of the reasons for improper visual examination is that physicians are not familiar with distinguishing childhood glaucoma owing to the rare nature of this disease^10^. Uncooperativeness of the children at examination also results in further delay in diagnosis. This deep-learning model allows clinicians to make non-contact diagnoses of childhood glaucoma with comparable accuracy to specialists, which may contribute to deciding on a referral to a specialist without delay.

The detection rate of childhood glaucoma largely depends on specific findings. Corneal opacity has been thought to be the most noticeable finding for caregivers and physicians^6,26^, and it was consistent with the good detection rate by both human (79%) and AI (96%) graders in this study. In contrast, the human detection rate of childhood glaucoma was considerably low with a transparent cornea (34%); however, AI showed better classification (72%). These results indicate that medical practitioners are capable of detecting corneal opacity solely by visual inspection but do not accurately detect periocular changes. As shown in the GradCAM heatmap of the periocular area, the deep-learning model likely compensates for physicians’ weakness and helps determine the indications for a full examination, including accurate measurement of corneal diameter using a ruler with or without general anesthesia^27,28^.

The background prevalence of the disease types in the dataset was similar to that reported in epidemiological studies^2,29,30^. The most common type of glaucoma was primary congenital glaucoma (31%), followed by anterior segment dysgenesis (22%), retinopathy of prematurity (14%) and Sturge–Weber syndrome (14%). In contrast, our dataset has several characteristics. First, our dataset did not include juvenile glaucoma with an onset of 4 years or older because we included only patients with periocular appearance change; corneal enlargement rarely occurs due to increased scleral rigidity^7^. Secondly, our dataset included more corneal diseases and fewer aphakic glaucomas than epidemiological frequency, possibly due to the disease specialties in our facility.

The end-to-end deep-learning model for childhood glaucoma classification is similar to other facial diagnostic systems, such as strabismus^12^, Turner syndrome^20^, and Down syndrome^21^. However, the development of the model is challenging for several reasons. First, childhood glaucoma is a rare disease, and the dataset included only 35 patients, while the above studies used 3829, 170, and 148 patients^12,20,21^. Secondly, 43.9% of the patients with glaucoma had strabismus. To cope with this overlap, we included the same percentage of strabismus patients in the control set and applied group-and-stratified fivefold cross-validation, in which each fold contained an equal percentage of strabismus and did not contain the same patients. Age was also matched between the groups to improve predictive accuracy.

We used RepVGG architecture for deep learning model in this study. RepVGG adopts structural re-parameterization to decouple a multi-branch shape for training with a plain architecture for interference^31^, which enables high accuracy and short prediction time at the same time. RepVGG achieved over 80% top-1 accuracy on ImageNet, comparable to state-of-art models such as EfficientNet^31^. In addition, it ran 83% faster than ResNet-50 and 101% faster than ResNet-101, while still providing a higher accuracy. These features would be very useful in actual clinical use when socially implemented.

The present deep-learning model has several drawbacks. This model is not robust to pediatric corneal diseases other than glaucomas, such as Peter’s anomaly, corneal dystrophies, and sclerocornea, because images of such patients were not included in this dataset. However, we believe that this is acceptable for screening patients who need a quick referral to a specialist. As well, an external dataset was not available to evaluate the generalized performance, which needs to be confirmed in the future. In addition, the dataset included only Japanese subjects. Further data collection needs to be applied to other ethnic groups. Finally, specialists in “childhood glaucoma” were not included in the human graders because they participated in the collection of images. Therefore, it remains unclear whether the diagnostic performance of AI is comparable to that of “true specialists” in this field.

Future application of this deep-learning model is to integrate with other deep learning modalities. For example, childhood glaucoma is associated with not only change in shape in the anterior segment of the eyes, but also with in the shape in the optic disc. The combination of the existing deep learning models to detect glaucomatous change of the optic disc during fundus examination may increase the accuracy of diagnosis^21–23^.

In conclusion, the deep-learning system presented in this study demonstrated comparable performance in detecting childhood glaucoma as compared to pediatric ophthalmologists or glaucoma specialists who performed the diagnosis based on visual examination. This system has a potential in assisting medical practitioners in making referral decisions to specialists by improving the accuracy and efficiency of glaucoma diagnosis and treatment in children.

## Methods

### Study design

This study was a retrospective diagnostic cross-sectional study from the medical records and imaging archives of Osaka University Hospital (OUH). This study followed the tenets outlined in the Declaration of Helsinki and was approved by the Institutional Review Board (IRB) of Osaka University Hospital (identifier: 19492-T1). For children whose photographs are included in the figures, informed consent was obtained from the parents or legal guardians of all child participants for both their participation in the study and for the release of information and images in open access publications.

### Datasets

In this cross-sectional study, we collected 65,534 periocular photographs of 8125 patients from the imaging archive of the Department of Ophthalmology, OUH, from November 2000 to September 2021. The images were carefully reviewed by multiple ophthalmologists, and those with characteristic facial features of childhood glaucoma (opacity and/or enlargement of the cornea and/or eyeball) were included in the glaucoma group. For the control group, primary gaze photographs of patients aged 0–10 years were selected from among patients without childhood glaucoma. Since many childhood glaucoma patients had strabismus, we also included esotropia and exotropia patients in the control group in the same proportions as the glaucoma group. The age and sex distributions were adjusted.

The exclusion criteria were eyelid diseases, hereditary facial deformities, and corneal diseases. We also excluded photographs with tilted faces, wearing glasses, or after eyelid or squint surgeries. All photographs were taken 1 m from the subject using a commercially available camera (D850; Nikon Inc., Tokyo, Japan) with a ring flash. The original images were in Jpeg format and the size was 2034 × 1536 pixels.

### Experimental setup

**Figure 3:**
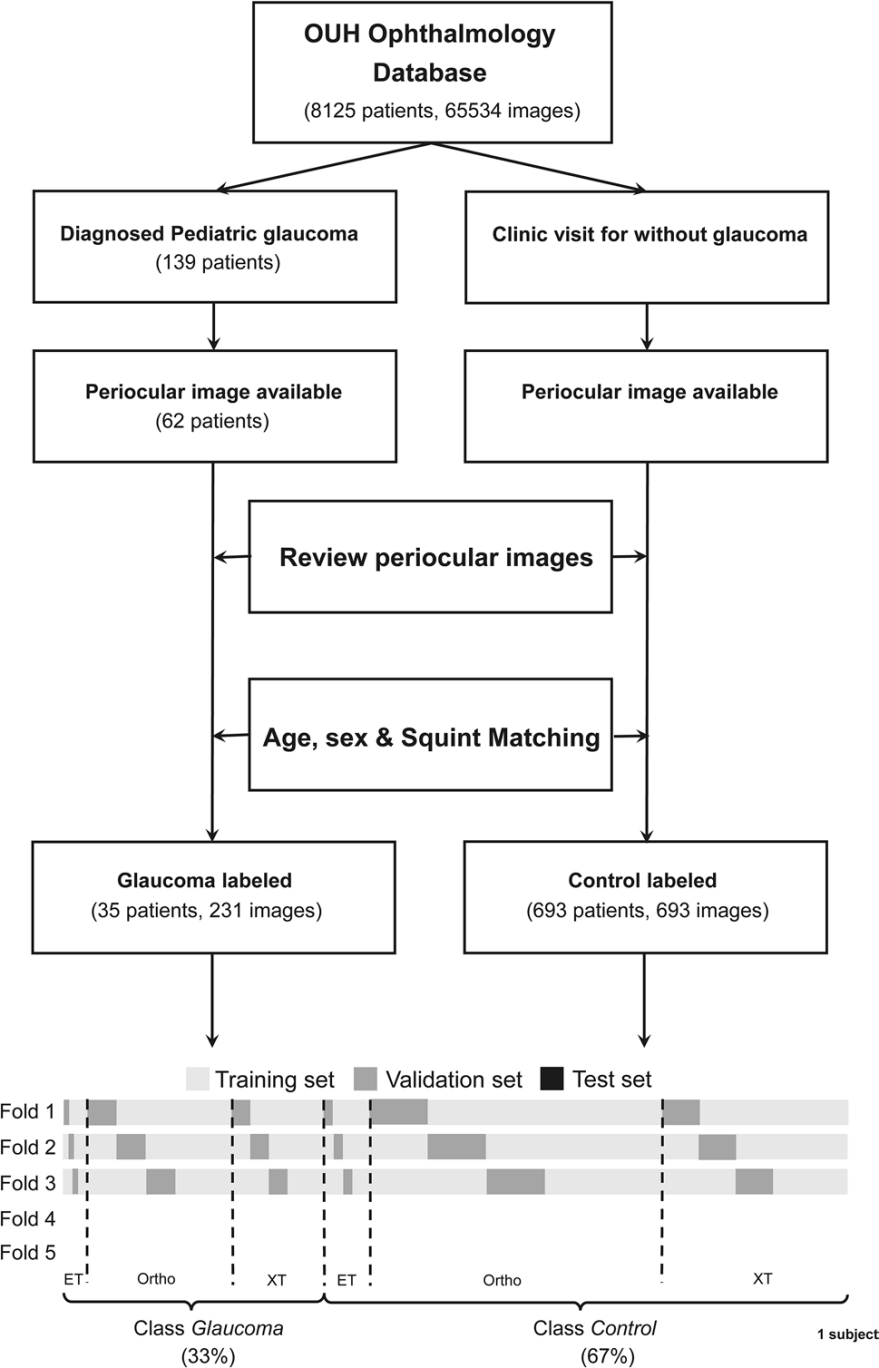
Diagrams of image dataset collection and pre-processing for detecting childhood glaucoma using deep learning.

In our methodology, we used fivefold stratified group and a leave-one-subject-out cross-validation approach. In brief, all images of a single subject in glaucoma or control group were first used as the test set. Next, the remaining photographs were randomly divided into a training set and a validation set in an 80:20 ratio, so that the proportion of esotropia, orthotropic, and exotropia was the same (1:5:4) between the divided datasets so that images in a single subject did not span multiple datasets (Fig. 3).

We implemented a deep-learning algorithm using the PyTorch framework (version 1.10.0). The models were trained on a Windows 10 Pro (version 21H2, build 19044.2486) operating system (Microsoft Corporation, Redmond, WA, USA) with an Intel Xeon E-2276M CPU, 32 GB RAM, and NVIDIA Quadro RTX 5000 16 GB GPU. The study was conducted using the Windows 10 operating system. The performance of the algorithm was measured using diagnostic parameters including accuracy, sensitivity, specificity, F-value, and area under the receiver operating curve. We then compared the diagnostic accuracy of the deep-learning algorithm with that of two glaucoma specialists and two pediatric ophthalmologists in the ophthalmology department.

### Deep-learning algorithms

**Figure 4:**
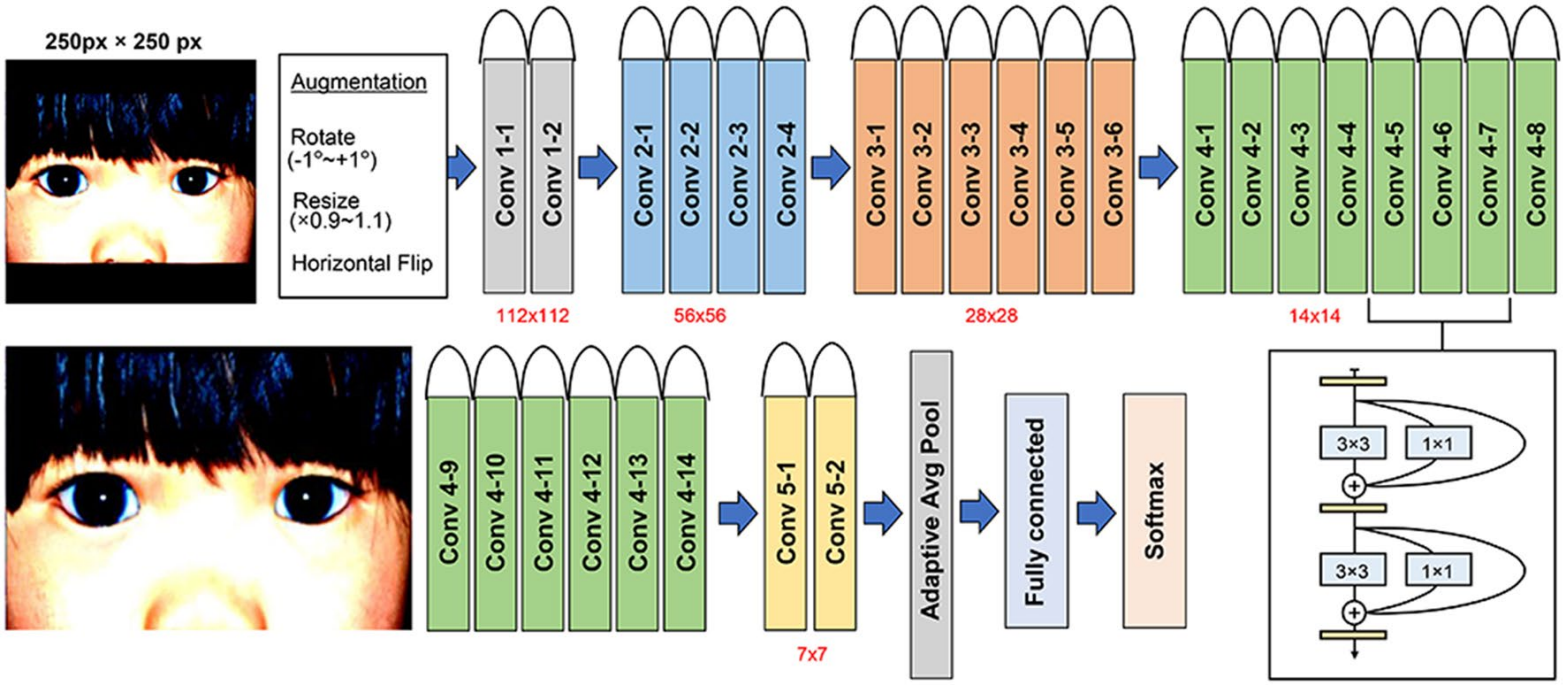
The structure of the convolutional neural network (RepVGG-A2). The input of the network is 224 × 224 in size.

We applied transfer learning based on the DCNNs architecture that was pre-trained on ImageNet (1000 object categories with more than 1 million images). The applied DCNNs were RepVGG-A2^31^. To make use of the pre-trained DCNN model, the images were formatted as a square with the long side as one edge and the white space filled in black (Fig. 4) to a size of 250 × 250 pixels. Data augmentation was performed to increase the number of images, including random horizontal flip, random rotation (± 1 degree), random resize (× 0.8 to × 1.1), and crop to 224 × 224 × 3 to adjust the pre-trained network. Images were then normalized and standardized based on ImageNet values to improve uniformity. Randomization techniques were utilized in the training process to help the model learn more variations in the images. For training, Adabelief optimizer was selected for training based on its fast convergence and optimal performance, as determined by a pilot evaluation that included SGD, RMSprop, Adam, AdamW, and Adabelief^32^. The learning rate was set to 0.001 after evaluating the impact of different learning rates (0.1, 0.01, 0.001, and 0.0001). The network was trained for 200 epochs using a batch size 16 and early stopping was applied when the validation loss did not decrease for 10 epochs (Table 5).

**Table 5:** The hyperparameters used in this work.

Parameter	Value
Model	RepVGG-A2
Optimizer	Ranger-Adabelief
Learning rate	1e−3
Epsilon	1e−12
Loss function	Cross entropy loss
Betas	(0.9, 0.999)
Epochs	200
Batch size	16
Early stopping	10

### Performance interpretation and statistics

All statistical analyses were performed using the scikit-learn library in Python (version 3.8.6; Python Software Foundation, Beaverton, OR). The Welch’s t-test was used to compare the ages of glaucoma patients and controls. The chi-square test was employed to compare the distribution of sex and eye position between the two groups. Statistical significance was defined as a p-value of less than 0.05.

We evaluated the performance of the DCNN algorithm by determining the area under the receiver operating characteristic (ROC) curve (AUC). The diagnostic performance was assessed per image by calculating the accuracy, sensitivity, specificity, positive predictive value, F-measure, and AUC. F-measure was defined as (2 × sensitivity × positive predictive value)/(sensitivity + positive predictive value)^33^. In addition, we ensembled fivefold prediction results and evaluated the prediction performance when a positive result of at least onefold was defined as positive. For each analysis, the classification threshold was fixed at 0.5.

To provide visual explanations for our deep-learning algorithms, we used gradient-weighted class activation mapping (Grad-CAM)^34^.

### Comparison of the deep-learning algorithms with human examiners

We randomly selected images of 35 glaucoma and 35 control subjects to compare the diagnosis of the deep-learning algorithm with that made by clinicians. Two glaucoma specialists (T. O. and S. U.) and two pediatric ophthalmologists (T.F and H.S) who were blinded to the data collection procedures were instructed to decide whether each test image corresponded to the glaucoma group or the control group, independently. They were informed that all images in the glaucoma group included characteristic findings of childhood glaucoma, such as enlargement of the globe and/or corneal opacity. The prediction of the deep-learning model was determined by a majority vote of the prediction of all fivefold models.

## Data Availability

The main data supporting the results of this study are available in this manuscript. The raw datasets from Osaka University Hospital cannot be made available because of hospital regulation restrictions and patient privacy concerns. Some anonymized data are available for research purposes from the corresponding authors upon reasonable request.
